# Role of hepatic cytochrome P450 enzymes in the detoxication of aristolochic acid I; effects on DNA adduct, mutation, and tumor formation

**DOI:** 10.1186/s41021-015-0010-z

**Published:** 2015-07-30

**Authors:** Yang Luan, Guozhen Xing, Jin Ren, Jun Gu

**Affiliations:** Hongqiao International Institute of Medicine, Shanghai Tongren Hospital/Faculty of Public Health, Shanghai Jiao Tong University School of Medicine, 227 South Chongqing Road, Shanghai, 200025 China; Center for Drug Safety and Evaluation Research, State Key Laboratory of New Drug Research, Shanghai Institute of Materia Medica, Chinese Academy of Sciences, Shanghai, 201203 China; Wadsworth Center, New York State Department of Health, Empire State Plaza, Box 509, Albany, NY 12201-0509 USA

**Keywords:** Aristolochic acid I (AAI), Hepatic cytochrome P450 (CYP), *gpt* delta mice, DNA adduct, Gene mutation assay, Tumorigenesis

## Abstract

**Introduction:**

Hepatic cytochrome P450s (CYPs) play an important role in the metabolism of plant carcinogen, aristolochic acid I (AAI). In the present study, we employed hepatic NADPH-cytochrome P450 reductase null (HRN) *gpt* delta transgenic mice to investigate the role of hepatic CYPs in the metabolism of AAI. DNA adduct formation, gene mutation, and tumor induction in the liver and kidneys were analyzed. Pharmacokinetic analyses were performed and tissue levels of AAI were determined.

**Results:**

Pretreatment with β-naphthoflavone in wild type *gpt* delta transgenic mice (BNF-WT mice) could increase the rate of clearance of AAI in blood and tissues, and decrease the formation of AAI-DNA adducts in kidney. In contrast, there was reduced clearance of AAI in HRN *gpt* delta mice, which showed increased concentration of AAI in tissues and increased levels of DNA adducts. The mutant frequencies of *gpt* gene, induced by AAI, in the kidneys of HRN *gpt* delta mice were significantly higher than that in WT mice. In the tumor induction assay, after treatment for 2 months with daily doses of 5 mg/kg AAI, mice were kept under observation for 7 months. During this period, papillomatous changes occurred in the forestomach of both WT-AAI mice and HRN *gpt* delta-AAI mice. Squamous cell carcinomas were found in the forestomach of 2 HRN *gpt* delta-AAI mice, which had also metastasized to other tissues. In addition, adenomas were found in 2 of 8 HRN *gpt* delta-AAI mice, in the absence of squamous cell carcinomas.

**Conclusion:**

These results indicated that the main role of hepatic CYPs is to aid in the excretion of AAI, and to protect the target organs against AAI induced DNA adduct formation, mutagenesis, and tumorigenesis.

**Electronic supplementary material:**

The online version of this article (doi:10.1186/s41021-015-0010-z) contains supplementary material, which is available to authorized users.

## Introduction

Aristolochic acid (AA), a naturally occurring nephrotoxin and carcinogen, derived from Aristolochia plant species, is associated with aristolochic acid nephropathy (AAN) [1, 2], Balkan endemic nephropathy (BEN) [3], and their urothelial malignancies [4]. Herbal remedies containing species of the genus Aristolochia and the AA mixture were classified as carcinogenic to humans (Group 1) by the International Agency for Research on Cancer [5].

**Fig. 1:**
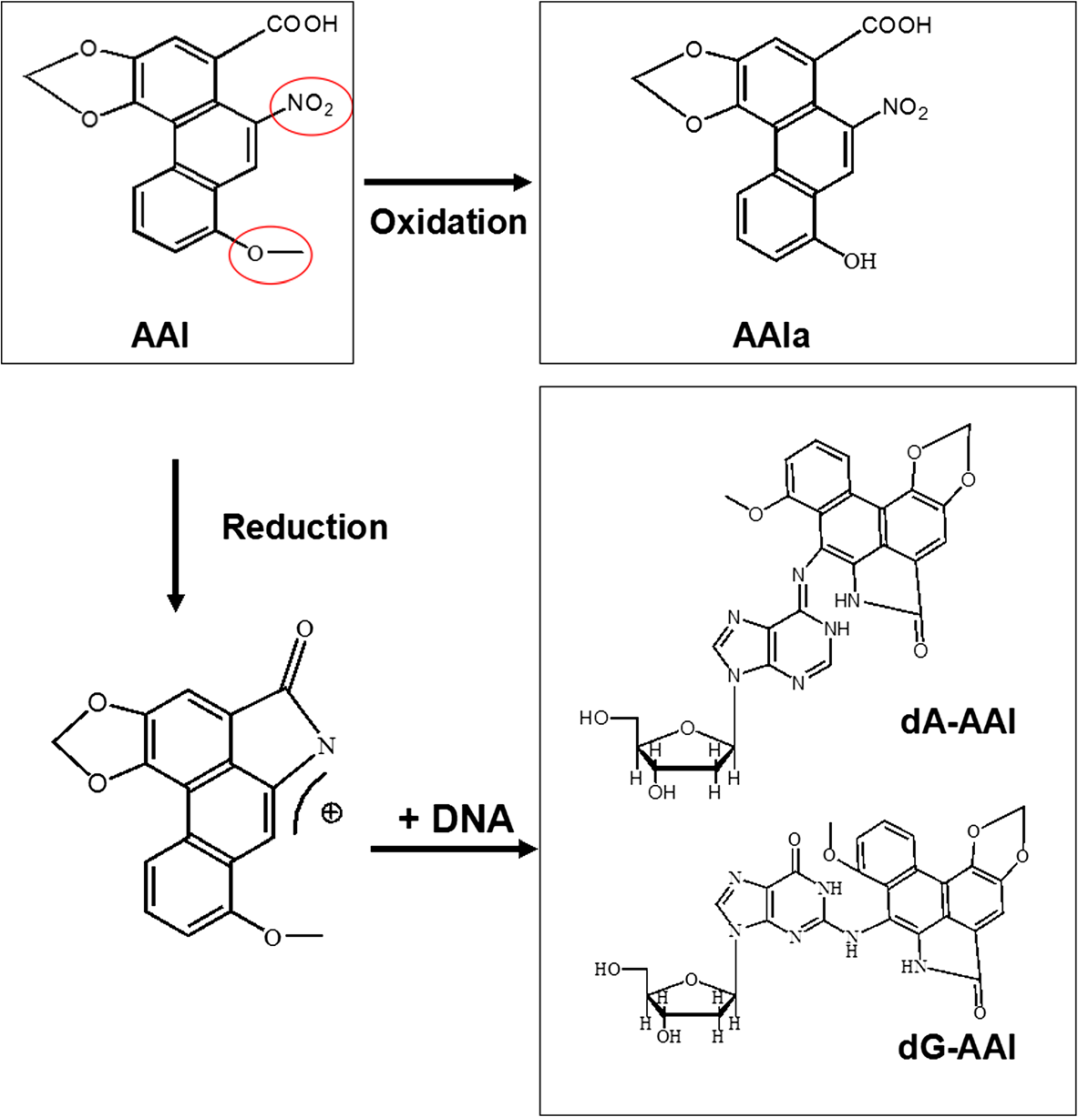
The balance between oxidative detoxication and reductive activation of aristolochic acid I (AAI). Detoxification of AAI by O-demethylation to generate AAIa, and bio-activation of AAI by nitroreduction to form DNA adducts including dA-AAI and dG-AAI. dA-AAI, 7-(deoxyadenosin-N6-yl) aristolactam I; dG-AAI, 7-(deoxyguanosin-N2-yl) aristolactam I; AAIa, 8-OH-aristolochic acid I

The toxic effects of aristolochic acid I (AAI), a major component of AA, have been well-studied. Metabolism of AAI results in nephrotoxic and carcinogenic effects (Fig. 1).

Demethylation of AAI to 8-OH-aristolochic acid I (AAIa) is believed to be a detoxification reaction, where AAIa is much less toxic and easily excreted in urine compared with AAI [6–8]. In contrast, nitroreduction of AAI is believed to be the major metabolic activation pathway, which potentiates the carcinogenic effect. Reduction of a nitro group of AA will form reactive cyclic nitrenium ions, which can interact with the exocyclic amino groups of deoxyadenosine and deoxyguanosine, resulting in the preferential formation of purine adducts 7-(deoxyadenosin-N 6-yl) aristolactam I (dA-AAI) and 7-(deoxyguanosin-N 2-yl) aristolactam I (dG-AAI) [9]. The predominant AA-DNA adduct dA-AAI is a mutagenic lesion resulting in A:T to T:A transversions in the p53 gene of urothelial tumors from AAN and BEN patients, and in codon 61 of H-Ras gene from AAI-treated rodents [10–12]. The cytochrome P450 (CYP) superfamily plays a pivotal role in both detoxification and activation of AAI. *In vitro* studies showed that AAI is reduced, instead of oxidized, at low oxygen concentrations, whereas under aerobic conditions AAI is oxidized to generate AAIa, suggesting the toxicity of AAI may depend on the balance between oxidation and reduction [13]. Since *in vitro* studies are limited by the absence of factors, that are important *in vivo*, such as the route of administration, coupling to phase-II xenobiotic metabolizing enzymes, and tissue-specific expression of CYPs, *in vivo* studies could provide relevant information on the actual process of AAI metabolism. In our previous study, we used conditional hepatic NADPH-cytochrome P450 reductase null mice (HRN mice), and demonstrated that hepatic CYPs detoxify AA through demethylation thus completing the detoxification cycle, and protecting kidneys from AA-induced mice kidney injury [14, 15]. Others studied the role of CYPs in AAI activation and the formation of AAI-DNA adducts in Cyp1a1(−/−), Cyp1a2(−/−) [16], Cyp1a1/1a2(−/−) mice [17], HRN mice [18], and humanized hCYP1A mice [19]. Since carcinogenesis is a multistep process, there is no single toxicological end point that is associated with complete carcinogenic potency. Risk assessment of a chemical should involve multistep analysis of carcinogenesis, and thus, the role of CYPs in AAI-induced mutagenesis and carcinogenesis *in vivo* still needs to be confirmed.

We previously reported the generation of an HRN *gpt* delta mouse model, by crossing HRN mice with *gpt* delta transgenic mice [20], which was a useful tool for investigating the role of hepatic CYPs in the metabolism of genotoxic carcinogens. In the present study, we analyzed the formation of DNA adducts, mutant frequencies of *gpt* gene as well as mutation spectrum characteristics, tumor induction in AAI- treated HRN *gpt* delta mice, combined with pharmacokinetic analysis and tissue level detection of AAI. Among the P450 superfamily, CYP1A is known to be involved in activation/detoxification of a variety of procarcinogens, in the present study, we pretreated animal with a non-carcinogen CYP1A inducer β-naphthoflavone (BNF) to clarify the role of hepatic CYP1A in the bioactivation of AAI by the detection of DNA adducts level. We finally determined the effect of hepatic CYPs on carcinogenesis, induced by AAI. We demonstrated that hepatic CYPs play a major role in completing the detoxification cycle and protect the target organs from AAI-induced genotoxicity.

## Materials and methods

### Chemicals

AAI (96 %) was obtained from Sigma-Aldrich Co. LLC. (St. Louis, MO, USA). Aristolactam I (ALI) was obtained from Shanghai ChemPartner Co. Ltd. (Shanghai, China). 2′-Deoxyadenosine (dA), 2′-deoxyguanosine (dG), DNase I, and phosphodiesterase I were obtained from Sangon Biotech Shanghai Co. Ltd. (Shanghai, China). Alkaline phosphatase was obtained from Sigma-Aldrich Co. LLC. (St. Louis, MO, USA). HPLC-grade methanol and acetonitrile were obtained from MSD Sharp & Dohme Corp. (Munchen, Germany). Other reagents were analytically pure.

### Animal use and treatment

Generation and characterization of HRN *gpt* delta mice were reported previously. The Shanghai Animal Care and Use Committee approved all animal experiments [Certificate No. SCXK (Shanghai) 2002–0010]. Animals were acclimatized in specific pathogen-free rooms with the temperature at 20 to 26 °C, humidity at 30 to 70 %, and a 12 h light/dark cycle for at least 1 week. Regular laboratory chow and filtered tap water were allowed, as per requirements.

#### AAI-DNA adduct analysis

Wild type (WT) mice were pretreated with a single intraperitoneal injection of BNF (BNF-WT) at 80 mg/kg in corn oil, once daily for 3 days, before injection of AAI. Male WT, BNF-WT, and HRN *gpt* mice were intragastically treated with AAI at a dose of 15 mg/kg for 2 days, then killed 24 h following the final treatment, and kidneys and livers were removed and stored at −40 °C, until further analysis for AAI-DNA adducts [20].

#### Mutation assay

Male WT *gpt* and HRN *gpt* delta mice, at the age of 8 weeks, were intragastically treated with 15 mg/kg AAI, once a week, for 4 consecutive weeks. One week after the final treatment, the mice were killed by cervical dislocation. Kidneys and livers were removed and quickly frozen in liquid nitrogen, then stored in a deep freezer at −70 °C until further analysis.

#### Tumor induction assay

Thirty-nine female WT *gpt* delta mice and 35 female HRN *gpt* delta mice used in this study were divided into 4 groups: WT *gpt* control, WT *gpt*-AAI, HRN *gpt* control, and HRN *gpt* delta-AAI. AAI was dissolved in 1 % NaCO_3_. Mice were dosed orally with 5 mg/kg AAI for 2 months. Mice were then killed for histological examination after a 4- and 7-month recovery.

### Determination of the levels of AAI and metabolites in blood, liver, and kidney

Blood samples (20 μl each) were collected by tail bleeding at 5, 10, 20, 30, 45, 60, 90, 120, 180 min and tissue samples were collected at 30, 60, 120 and 180 min after a single oral dose (15 mg/kg) of AAI (n = 4 per group) to male mice. Collected samples were further processed as described previously [21]. Aliquots of the final supernatants were analyzed and quantified, for the levels of AAI and its reductive metabolites aristolactam I (ALI) by HPLC. The identity of AAI and associated metabolites were confirmed with previously described standards.

### Quantitative analysis of AAI–DNA adducts in liver and kidney of mice

The isolation of total genomic DNA, from kidney and liver, was carried out by the phenol/chloroform method. DNA samples were digested and enriched by ethyl acetate. DNA adducts were detected by the liquid chromatography-electrospray ionization-multi-stage-mass spectrometry (LC-ESI-MS–MS) method, reported previously [23]. Deoxyadenosine-AAI (dA-AAI) (external standard) and tolbutamide (internal standard) were used to quantify the concentration of AAI-DNA adducts, and deoxyadenosine was used to quantify the concentration of normal deoxynucleosides.

### Mutation assay

Mutation assay of *gpt* gene was performed using the method, reported previously [22]. Briefly, genomic DNA was extracted from kidneys and liver, and lambda EG10 DNA (48 kb) was rescued as phages by *in vitro* packaging. Packaged phages were incubated with *Escherichia coli* YG6020. Infected cells were mixed with molten soft agar and poured onto agar plates containing chloramphenicol and 6-Thioguanine (6-TG). To determine the total number of rescued plasmids, infected cells were also poured onto plates containing chloramphenicol without 6-TG. The plates were then incubated at 37 °C. Positively selected colonies were counted on day 3 and collected on day 4. The mutant frequencies were calculated by dividing the number of *gpt* mutants by the number of rescued phages. *gpt* gene of mutant colonies were sequenced. Duplicate mutations at the same site within an individual tissue of *gpt* gene were excluded to account for colonial expansion of sibling mutations. The data obtained from the study were expressed as mean ± standard deviations. Dunnett’s test, after one-way ANOVA and Student’s *t*-test, was used to evaluate the differences. Mutational spectra were compared using the computer program written by Cariello [23], for the Monte Carlo analysis developed by Adams and Skopek [24]. The calculated *p* values, which were lower than 0.05 were considered to indicate statistical significance.

### Tumor induction assay

Mice were killed by an overdose of carbon dioxide (CO_2_). After a recovery of 4 or 7 months, the mice were terminated, and kidney, liver, stomach, small and large intestine, urinary bladder, lungs, and spleen were excised and fixed in 10 % formalin for histological examination. Paraffin sections (3 μm) were prepared and stained with haematoxylin and eosin, and examined microscopically.

## Results

### Levels of AAI and associated metabolites in plasma and tissues

**Fig. 2:**
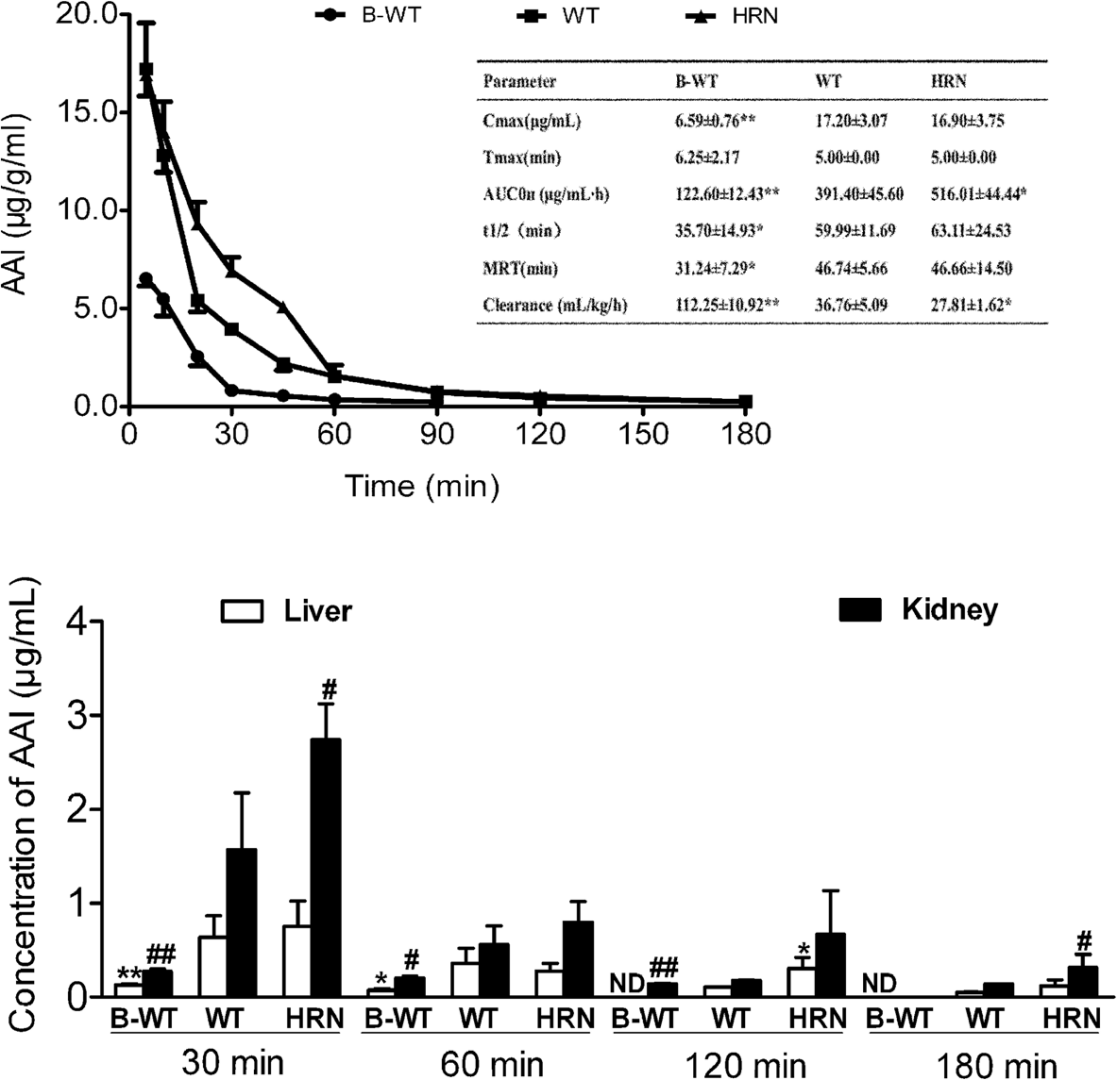
The concentrations of AAI in plasma and tissues in BNF-pretreated WT mice, WT mice and HRN *gpt* delta mice. Upper panel is serum levels of AAI. Mice were intragastrically treated with AAI at a single dose of 15 mg/kg, tail vein blood samples were collected from individual mice at eight time points after dosing for determination of AAI concentrations as described in Materials and Methods. Inset: pharmacokinetic variables in mice. Values presented as mean ± SD, n = 4. *, *p* < 0.05, **, *p* < 0.01, compared to WT mice. *MRT*, Mean Residence Time. Lower is the concentrations of AAI in the liver and kidney of mice. *B-WT*, wild-type mice were pretreated with β-naphthoflavone (BNF). *WT,* wild-type mice. *HRN,* hepatic Reductase Null *gpt* delta mice. #, *p* < 0.05, ##, *p* < 0.01, the concentration of AAI in kidney compared to WT mice. *, *p* < 0.05, **, *p* < 0.01, the concentration of AAI in liver compared to WT mice. N = 4

The contribution of hepatic CYPs to AAI metabolism was determined *in vivo* by comparing AAI levels in blood and tissues between the BNF-pretreated wild-type (BNF-WT) mice, wild-type *gpt* delta (WT) mice, and HRN *gpt* delta mice. The clearance of AAI was much slower in HRN *gpt* delta mice but faster in BNF-WT mice compared with WT mice (Fig. 2). The levels of AAI, in the liver and kidney, were determined at 30 min, 60 min, 120 min, and 180 min after the dosing of AAI at 15 mg/kg, using LC-MS/MS analysis. The levels of AAI were higher in HRN *gpt* delta mice than that in the WT group in both liver and kidney, which suggested that inactivation of hepatic CYPs reduced the clearance of AAI in HRN *gpt* delta mice. On the other hand, AAI levels in BNF-WT mice were lower than in WT mice, suggesting CYP1A may play a key role in the clearance of AAI.

### Formation of AAI-derived DNA adducts in liver and kidneys

In the detection of AAI-derived DNA adducts, mice were intragastically treated with AAI at 15 mg/kg for 2 days. DNA samples of kidney and liver were digested, enriched by ethyl acetate, and analyzed. Deoxyadenosine-AAI (dA-AAI) and dG-AAI were determined by the previously developed method of LC–MS–MS [22], and the response fell in the linear range between 4 ng/ml to 200 ng/ml. In liver tissue, AAI-DNA adducts from only HRN *gpt* delta-AAI mice can be detected, while BNF-WT-AAI and WT-AAI mice showed lower levels of adducts, where the response fell out of the linear range (Table 1). In kidney tissue, the total amount of AAI-DNA adducts formed in the HRN *gpt* delta-AAI mice was higher than those in BNF-WT-AAI mice and WT-AAI mice (*p* < 0.05). The DNA adduct pattern was different in the liver and kidney of HRN *gpt* delta-AAI mice, the major DNA adducts in the liver was dA-AAI, the level was about 1.5-fold higher than that of dG-AAI, whereas dG-AAI was predominant adducts in the kidney, showed a 1.5-fold higher level than dA-AAI. Pretreatment with BNF could reduce the level of AAI-DNA adducts, which is consistent with the reduction in the levels of ALI.

**Table 1:** Formation of AA-DNA adducts in the liver and kidney of *gpt* delta transgenic mice treated with AAI or AAII

AAI-derived DNA adducts	dA-AAI	dG-AAI	Total
	per 10^7^ dN	per 10^7^ dN	per 10^7^ dN
Liver			
B-WT	-	-	-
WT	-	-	-
HRN	15.92 ± 4.46	10.38 ± 4.86	26.29 ± 9.22
Kidney			
B-WT	029.94 ± 11.46	24.02 ± 6.30	053.96 ± 16.52
WT	58.09 ± 9.50	68.68 ± 6.93	126.77 ± 12.28*
HRN	101.59 ± 45.30	152.15 ± 26.52	253.75 ± 38.08*

*B-WT* wild-type mice were pretreated with β-naphthoflavone (BNF); *WT* wild-type mice, *HRN* hepatic Reductase Null *gpt* delta mice

Values presented are mean ± SD, n = 5, dN, deoxynucleosides.*, *p* < 0.05 compare with WT mice. -, not detected

### Mutant frequencies of *gpt* induced by AAI in the liver and kidneys

**Fig. 3:**
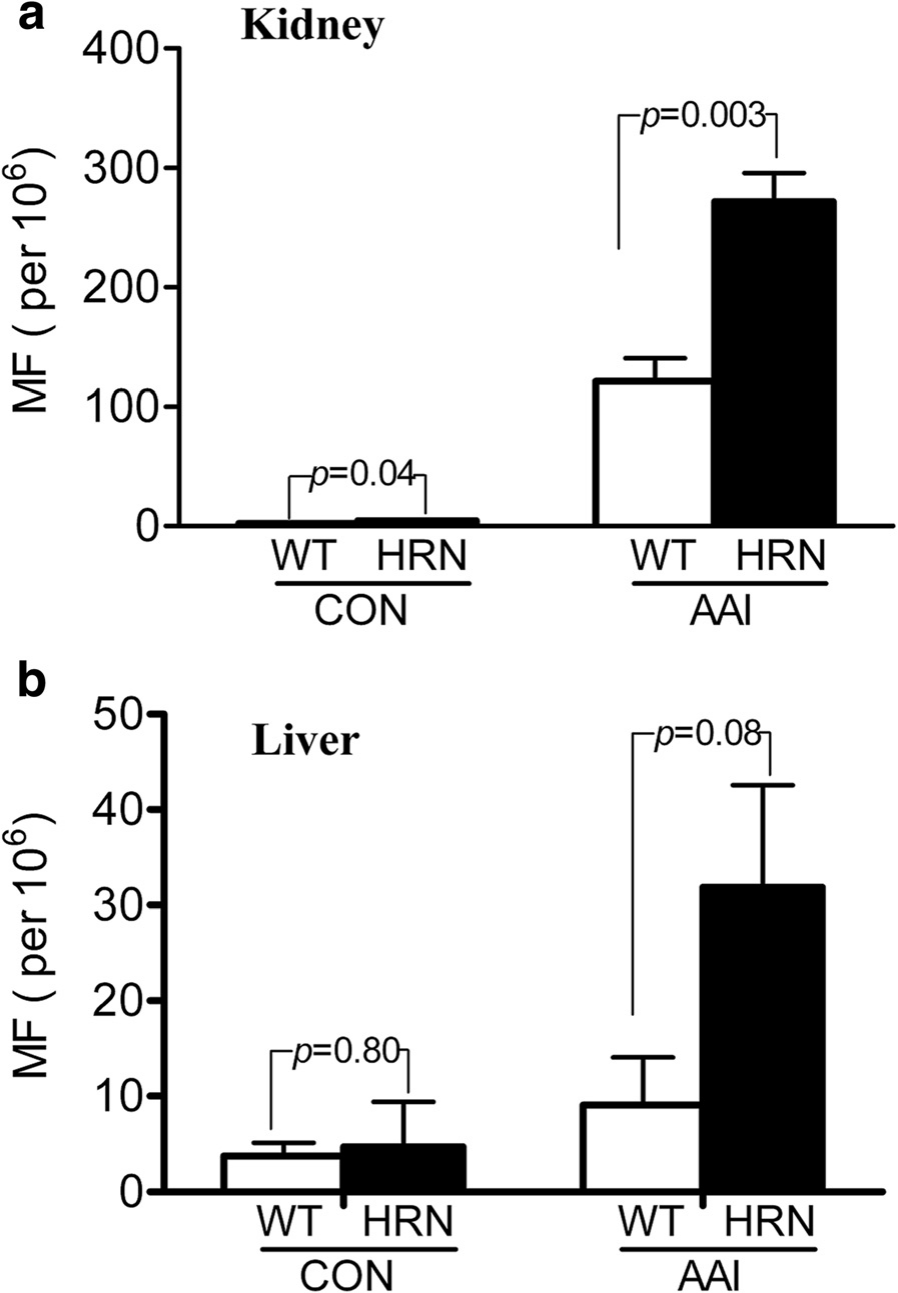
Mutant frequencies of *gpt* induced by AAI in the kidney and liver of mice. Eight-week-old, male, WT and HRN *gpt* delta mice were administrated AAI once a week for 4 weeks. One week after the last dose, the mice were euthanized. The liver and kidney were taken and quickly frozen in liquid nitrogen, then kept in freezer at −70 °C until being analyzed. **a**, the mutant frequencies of *gpt* gene in the kidney of mice. The mutant frequencies of *gpt* in kidney for WT Control, HRN *gpt* delta Control, WT-AAI and HRN-AAI were 2.49 ± 0.74 × 10^−6^, 4.52-1.37 × 10^−6^, 121.27 ± 38.48 × 10^−6^ and 271.96 ± 47.59 × 10^−6^ respectively. **b**, the mutant frequencies of *gpt* in the liver of mice. The mutant frequencies of *gpt* in liver for WT Control, HRN Control, WT-AAI and HRN-AAI were 2.03 ± 1.78 × 10^−6^, 5.71 ± 2.71 × 10^−6^, 9.26 ± 9.73 × 10^−6^ and 32.81 ± 32.05 × 10^−6^ respectively. Values presented are the mean ± SD, N = 5

To calculate the MFs of *gpt* gene, induced by AAI, we analyzed 205 500 to 843 000, and 172 500 to 1 027 500 chloramphenicol (Cm)-resistant colonies derived from the rescued phages of kidneys and liver, respectively. The frequency of spontaneous mutations in HRN *gpt* delta-AAI mice was not significantly different when compared to control littermates; their values were 5.71 ± 2.71 × 10^−6^ compared to 2.03 ± 1.78 × 10^−6^ in the liver, and 4.52 ± 1.37 × 10^−6^ compared to 2.49 ± 0.74 × 10^−6^ in the kidneys, within a historical acceptable range. The MFs of kidney and liver in HRN *gpt* delta-AAI mice were 271.96 ± 47.59 × 10^−6^ and 32.81 ± 32.05 × 10^−6^, which were 2-fold and 3-fold higher than those of WT-AAI mice, ie, 121.27 ± 38.48 × 10^−6^ and 9.26 ± 9.73 × 10^−6^, respectively (Fig. 3). We also determined the mutation spectra of *gpt* gene in the kidneys and livers of HRN *gpt* delta-AAI mice and WT-AAI mice (Additional file 1: Tables S1, S2). The main mutation was A:T to T:A transversion, whereas G:C to A:T was the dominant mutation in the untreated mice (*p* > 0.05), which was consistent with the current understanding of AAI-induced mutagenesis and reported data.

### Preneoplasia and neoplasia induced by AAI

**Fig. 4:**
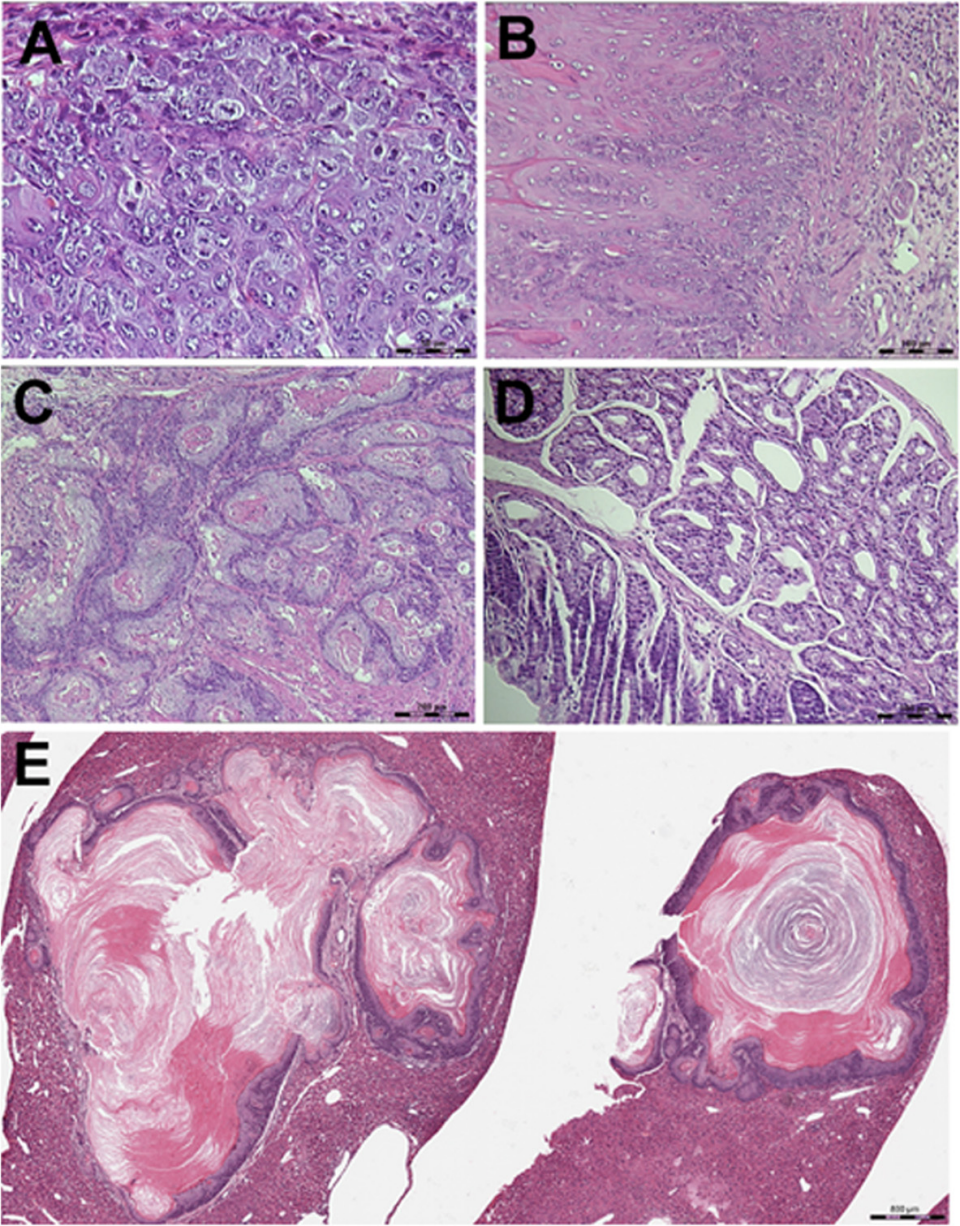
Histopathological features of neoplastic changes in WT *gpt* and HRN *gpt* delta mice induced by AAI. **a**, the papilloma with vacuolated cytoplasm, irregular nuclei and numerous mitoses, mainly atypical forms was found in one WT *gpt* mouse (HE × 400). **b**, the papilloma penetrated through muscularis mucosae found in forestomach of one HRN *gpt* mouse(HE × 200). **c**, Squamouse cell carcinoma was found in one HRN *gpt* delta mouse which had penetrated through stomach to invade esophagus (HE × 100). **d**, Adenoma was found in one HRN *gpt* delta mouse without squamous cell carcinomas (HE × 200). **e**, a metastasis was present in the liver of one HRN *gpt* delta mouse in which squamouse cell carcinoma had invaded into esophagus (HE × 500)

To elucidate the contribution of hepatic CYPs to AAI induced carcinogenesis, tumor induction was determined following exposure to AAI at 5 mg/kg/day for 2 months. The survival rates of WT *gpt*-AAI mice and HRN *gpt* delta-AAI mice were 62.5 % (5/8) and 80.0 % (8/10) for recovery in 4 months, and 47.1 (8/17) and 42.1 % (8/19) for recovery in 7 months, respectively, whereas the survival rate of control groups (WT *gpt* and HRN *gpt*) was 100 %. No significant difference, in survival rate, was found between WT *gpt*-AAI and HRN *gpt* delta-AAI mice. All neoplastic findings are shown numerically in Table 2. After treatment for 2 months and recovery of 4 months, the squamous epithelium showed marked hyperplasia and hyperkeratosis in both WT *gpt*-AAI and HRN *gpt* delta-AAI mice. Papillomatous changes occurred in the forestomach in both WT *gpt*-AAI and HRN *gpt* delta-AAI mice (Table 2). Most papillomas, observed at some points, were coneshaped and bulging out of the epithelium against the submucosa without penetrating the muscularis mucosae, except one HRN *gpt* delta-AAI mouse, where the papilloma penetrated through the muscularis mucosae (1/8) (Fig. 4b). Papillomas with vacuolated cytoplasm, irregular nuclei, and numerous mitoses, mainly atypical forms were found in one WT *gpt*-AAI mouse (Fig. 4a). After recovery of 7 months, papilloma with signs of malignancy and penetration through muscularis mucosae or presentation of irregular nuclei were found in 2 WT *gpt*-AAI mice (2/8) and one HRN *gpt* delta-AAI mouse (1/8). Squamous cell carcinomas were found in 1 WT *gpt*-AAI mouse (1/8), which was within the stomach. Squamous cell carcinomas were found in 2 HRN *gpt* delta-AAI mice (2/8), which had penetrated through the stomach to invade the esophagus, glandular stomach, and other surrounding tissues (Fig. 4c), and 1 metastasis was present in the liver had invaded the esophagus (Fig. 4e). Histologically, tumors were identified as keratinized squamous cell carcinomas with epithelial pearl formation, irregular nuclei, and disappearance of normal tissue structure. Adenoma was found in the duodenum in 2 other HRN *gpt* delta-AAI mice (2/8) treated with AAI, in the absence of squamous cell carcinomas, after recovery of 7 months (Fig. 4d). No preneoplastic or neoplastic changes were detected in the control animals (WT *gpt* and HRN *gpt*) in either histological or macroscopic examinations. Karyomegaly, peritubular fibrosis, and basophilic atypical tubules were present in the kidneys of HRN/WT *gpt* delta mice, treated with AAI, after recovery of 4 and 7 months.

**Table 2:** Number of neoplsia in WT mice and HRN *gpt* delta mice treated with AAI for two months

Organ	Tumor	4 months recovery			7 months recovery		
		Con	WT	HRN	Con	WT	HRN
Forestomach	Papilloma with signs of malignancy	0	1	1		2	1
	Squamouse cell carcinoma	0	0	0		1	2
Duodenum	Adenoma	0	0	0		0	2
Mice with neoplasia		0/10	1/8	1/5	0/10	3/8	5/8

*WT* wild-type mice; *HRN* hepatic Reductase Null *gpt* delta mice

## Discussion

As several studies indicate, metabolism of AAI plays a very important role in the initiation and progress of aristolochic acid nephropathy (AAN) and Balkan endemic nephropathy (BEN) [19, 26–29]. Understanding which enzymes are involved in AAI activation and/or detoxification is important in the assessment of the susceptibility to this carcinogen. AAI could be demethylated to form AAIa, excreted individually or conjugated with glucuronide, acetate, or sulfate esters, which is the metabolic detoxification pathway of AAI. Human and rodent enzymes, CYP1A1 and CYP1A2, are the principal enzymes involved in the detoxification pathway *in vitro* and *in vivo*. AAI could also be activated by nitroreduction metabolism and generate an intermediate a cyclic nitrenium ion, which can covalently bind the exocyclic amino groups of DNA bases, NAD(P)H:quinone oxidoreductase (NQO1). CYP1A1 and CYP1A2 have been proven to be the main responsive enzymes, *in vitro* and *in vivo* [16–18]. Therefore, CYPs are involved in both detoxification and reductive activation of AAI. The present study has been designed to obtain an overall evaluation of the role of hepatic CYPs in the contrasting processes of detoxification or activation of AAI by measuring 3 toxic endpoints: DNA adduct formation, gene mutation, and tumor induction by using a novel HRN *gpt* delta mouse model.

In the present study, the levels of AAI, in blood, kidney, and liver, in HRN *gpt* delta-AAI mice were higher than those in BNF-WT-AAI mice and WT-AAI mice. The number of AAI-DNA adducts formed in the kidney and liver of HRN *gpt* delta-AAI mice were also higher than those in BNF-WT-AAI mice and WT-AAI mice. These results suggested that hepatic CYPs metabolize AAI, and reduce the exposure level and formation of AAI-DNA adducts *in vivo,* which were consistent with the results reported previously [21, 30]. We further determined the *gpt* mutant frequencies, mutation spectrum, and tumor induction to study the effects of hepatic CYPs on AAI-induced mutagenesis and tumorigenesis by using the HRN *gpt* delta-AAI mice. Our findings provide further substantial evidence that the main role of hepatic CYPs is to help AAI to be excreted, and to protect target organs against AAI-induced mutagenesis and tumorigenesis.

We previously reported HRN *gpt* delta mice could provide useful information on the metabolism of carcinogen [15]. It is indisputable that a direct measure of induced gene mutations is better than other genetic end points such as DNA damage. The formation of DNA adducts is a critical determinant of mutagenicity, however, the final mutational events depend also on DNA repair which can eliminate DNA adducts. In the present study, our data showed the level of dG-AAI was higher than dA-AAI in the kidney of AAI treated HRN-*gpt* mice, but the dominant mutation pattern was at A:T- > T:A in the kidney of AAI treated HRN-gpt mice (Additional file 1: Table S1), suggested the DNA repair efficiency might be different between dA-AAI and dG-AAI, which need a further study. Moreover, metabolic activation usually generates various intermediates from different pathways, which may pose difficulties for detection of DNA adducts, although our *in vivo* gene mutation assay will overcome this limitation. Furthermore, HRN *gpt* delta mice could detect deletion mutations by Spi- assay. The Spi- assay results showed that HRN *gpt* delta mice had higher MFs than WT mice, but the magnitude of increase for Spi- assay was lower than that of 6-TG selection (Additional file 1: Figure S1). Mutation spectrum analysis showed the predominant deletion was 1 to 2 base pairs, which occurred in “C” or “G” bases in *gam* gene (Additional file 1: Table S3), which was different from 6-TG selection. The mechanism of mutagenesis thus, needs to be further investigated.

Carcinogenic profiles are different between rats and mice dosing with AA in varied recipes [25, 31–33]. The papillomas and squamous cell carcinomas in forestomach and urinary bladder, adenomas, mesenchymal tumours and oncocytoma in kidney, carcinomas in renal pelvis et al. could be found in rats [31–33], whereas papillomas and squamous cell carcinomas in forestomach, carcinomas in lung, adenomas in kidney et al. could be found in miceafter administration of different doses of AA or AAI in different dosing durations [25]. The results indicated that the main carcinogenic profiles up to date for the mice are the papillomas and squamous cell carcinomas occurred in forestomach, the carcinomas in lung and the adenomas in kidney.

It was reported that the short-term high dose AA or AAI exposure could induce cancers and tumors in both rats and mice though the profile were somehow different. Mengs [25] found papillomatous changes occurred in the forestomach, squamous cell carcinomas were observed in all the animals, adenomas of the kidneys, carcinomas of the lungs, and haemangiomas of the uteri after treatment for 3 weeks with AA in daily doses of 5.0 mg/kg in NMRI mice which were kept under observation for approximately 1 year. Another study reported by Cui et al. [33] showed female Sprague–Dawley rats dosed with AAI at 50 mg/kg for consecutive three days could induce tumors in kidney. Based on this speculation, the dose at 5 mg/kg AAI was selected for our study and the dosing duration was also prolonged to 2 months (nearly 8 weeks) which was longer than Mengs’ study and was supposed to induce tumors or cancers earlier. The total durations for determining the tumorigenesis or carcinogenesis were 6 months and 9 months after the first administration which equivalent to the durations of 26 weeks and 37 weeks reported by Mengs [25]. Due to the high tumor incidence found in the Mengs’ study, the minimum group size should be ≥ 4. We aimed to obtain the comparable data between AAI-gpt and AAI-HRN-gpt to investigate if there were differences between them. The papilloma and squamous cell carcinomas were present in the forestomach in both, AAI-gpt and AAI-HRN-gpt mice. We found adenomas in the duodenum in 2 other AAI-HRN-gpt mice in the absence of squamous cell carcinomas. One squamous cell carcinoma was present in the liver of one HRN mouse, and had invaded the esophagus. The results indicated that the AAI-HRN-gpt mice showed a higher sensitivity for AAI tumorigenesis or the when CYPs activity is reduced in liver more tumors and of higher severity are induced in the extra-hepatic target tissues.

## Conclusion

By using HRN *gpt* delta mice, we demonstrated that although hepatic CYPs are involved in AAI-induced mutagenicity, the main role of CYPs is to help AAI to be excreted, and to protect target organs against AAI-induced DNA adduct formation, mutagenesis, and tumorigenesis.

## Additional file

Additional file 1: Figure S1. Spi- mutant frequencies of deletions induced by AAI in the kidney and liver of mice. Eight-week-old, male, WT and HRN *gpt* delta mice were administrated AAI once a week for 4 weeks. One week after the last dose, the mice were euthanized. The liver and kidney were taken and quickly frozen in liquid nitrogen, then kept in freezer at -70^o^C until being analyzed. **Table S1** Summary of independent mutations in the *gpt* gene of kidney from AAI treated and control mice. **Table S2** Summary of independent mutations in the *gpt* gene of liver from AAI treated and control mice. **Table S3** Summary of Spi- deletions in WT and HRN mice induced by AAI.
