# Supplementary material for: Role of hepatic cytochrome P450 enzymes in the detoxication of aristolochic acid I; effects on DNA adduct, mutation, and tumor formation
Source: Genes Environ. 2015 Jul 30;37:11. doi: 10.1186/s41021-015-0010-z (PMC4918019; doi:10.1186/s41021-015-0010-z)
Supplement: Additional file 1: Figure S1. — Spi- mutant frequencies of deletions induced by AAI in the kidney and liver of mice. Eight-week-old, male, WT and HRN gpt delta mice were administrated AAI once a week for 4 weeks. One week after the last dose, the mice were euthanized. The liver and kidney were taken and quickly frozen in liquid nitrogen, then kept in freezer at -70oC until being analyzed. Table S1 Summary of independent mutations in the gpt gene of kidney from AAI treated and control mice. Table S2 Summary of independent mutations in the gpt gene of liver from AAI treated and control mice. Table S3 Summary of Spi- deletions in WT and HRN mice induced by AAI. [file 41021_2015_10_MOESM1_ESM.doc]

**Suppl Fig S1**

**
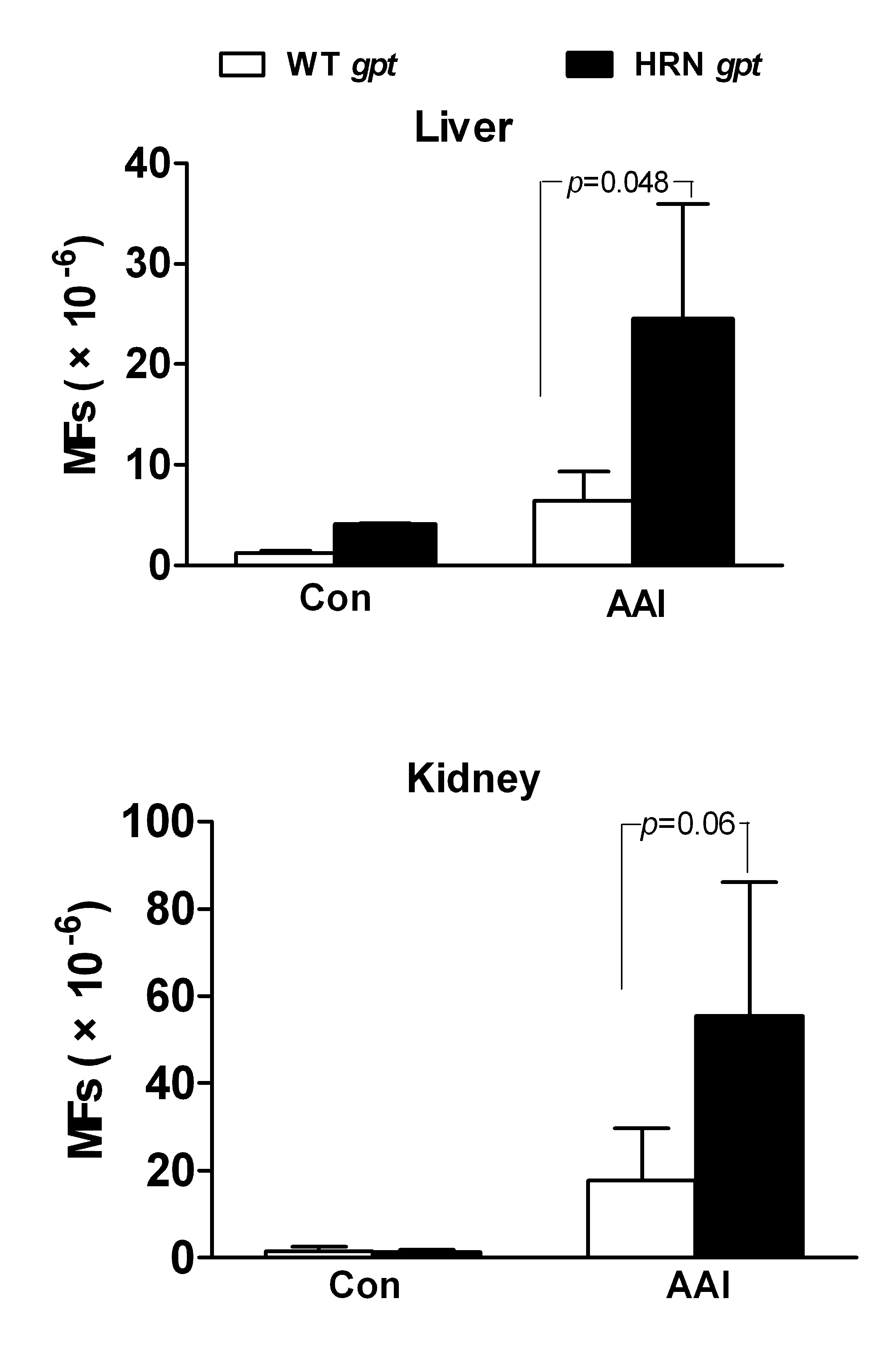
**

**Suppl Fig 1 Spi- mutant frequencies of deletions induced by AAI in the kidney and liver of mice.** Eight-week-old, male, WT and HRN *gpt* delta mice were administrated AAI once a week for 4 weeks. One week after the last dose, the mice were euthanized. The liver and kidney were taken and quickly frozen in liquid nitrogen, then kept in freezer at -70^o^C until being analyzed.

**Suppl Table 1 Summary of independent mutations in the *gpt* gene of kidney from AAI treated and control mice.**

| **Mutation type** | **WT-Con** | | | **HRN-Con** | | | **WT-AAI** | | | **HRN-AAI** | | |
| --- | --- | --- | --- | --- | --- | --- | --- | --- | --- | --- | --- | --- |
|  | **Mutants**  **(NO. at CpG site)** | | **%** | **Mutants**  **(NO. at CpG site)** | | **%** | **Mutants**  **(NO. at CpG site)** | | **%** | **Mutants**  **(NO. at CpG site)** | **%** | |
| Base substitution |  |  | |  |  | |  |  | |  | |  |
| Transitions |  |  | |  |  | |  |  | |  | |  |
| GC→AT | 8 (5 CpG) | 42.1 | | 4 (2 CpG) | 44.4 | | 4 (3 CpG) | 7.8 | | 9 (3 CpG) | | 15.3 |
| AT→GC | 1 | 5.3 | | 0 | 0.0 | | 0 | 0.0 | | 0 | | 0.0 |
| Transversions |  |  | |  |  | |  |  | |  | | 0.0 |
| AT→CG | 1 | 5.3 | | 0 | 0.0 | | 0 | 0.0 | |  | | 0.0 |
| AT→TA | 0 | 0 | | 4 | 44.4 | | 25 | 49.0 | | 32 | | 54.2 |
| GC→CG | 1 | 5.3 | | 0 | 0.0 | | 1 | 2.0 | | 2 | | 3.4 |
| GC→TA | 4 | 21.1 | | 1 | 11.1 | | 5 | 9.8 | | 2 | | 3.4 |
| Deletion | 2 | 10.5 | | 0 | 0.0 | | 7 | 13.7 | | 3 | | 5.1 |
| Insertion | 1 | 5.3 | | 0 | 0.0 | | 0 | 0.0 | | 0 | | 0.0 |
| Complex mutation^#^ | 1 | 5.3 | | 0 |  | | 9 | 17.7 | | 11 | | 18.6 |
| Total mutants | 19* | 100.0 | | 9 | 100.0 | | 51 | 100.0 | | 59 | | 100.0 |

^#^, Complex mutation, one mutant included more than two mutation spots.

*, 9 mutants from our own experiments, and 10 mutants from literature [21].

**Suppl Table 2 Summary of independent mutations in the *gpt* gene of liver from AAI treated and control mice.**

| **Mutation type** | **WT-Con** | | **HRN-Con** | | **WT-AAI** | | **HRN-AAI** | |
| --- | --- | --- | --- | --- | --- | --- | --- | --- |
|  | **Mutants**  **(NO. at CpG site)** | **%** | **Mutants**  **(NO. at CpG site)** | **%** | **Mutants**  **(NO. at CpG site)** | **%** | **Mutants**  **(NO. at CpG site)** | **%** |
| Base substitution |  |  |  |  |  |  |  |  |
| Transitions |  |  |  |  |  |  |  |  |
| GC→AT | 27 (5 CpG) | 46.6 | 2 (2 CpG) | 25.0 | 4 (3 CpG) | 30.8 | 1 (1 CpG) | 2.5 |
| AT→GC | 4 | 6.9 |  |  |  |  |  |  |
| Transversions |  |  |  |  |  |  |  |  |
| AT→CG | 0 | 0.0 | 0 | 0.0 | 0 | 0.0 | 1 | 2.5 |
| AT→TA | 4 | 6.9 | 2 | 25.0 | 5 | 38.5 | 32 | 80.0 |
| GC→CG | 2 | 3.4 | 0 | 0.0 | 1 | 7.7 | 2 | 5.0 |
| GC→TA | 7 | 12.1 | 0 | 0.0 | 1 | 7.7 | 0 | 0.0 |
| Deletion | 8 | 13.8 | 1 | 12.5 | 1 | 7.7 | 1 | 2.5 |
| Insertion | 1 | 1.7 | 1 | 12.5 | 0 | 0.0 | 0 | 0.0 |
| Complex mutation^#^ | 5 | 8.6 | 2 | 25.0 | 1 | 7.7 | 3 | 7.5 |
| Total mutants | 58* | 100.0 | 8 | 100.0 | 13 | 100.0 | 40 | 100.0 |

^#^,Complex mutation, one mutant included more than two mutation spots.

*, 9 mutants from our own experiments, and 49 mutants were from literature[22]

**Suppl Table3 Summary of Spi- deletions in WT and HRN mice induced by AAI**

| **Group** | **Tissue** | **Mutant No.** | **Position** | **Change** |
| --- | --- | --- | --- | --- |
| WT *gpt*-AAI | Kidney | 4 | 267 | ATCGA→ATGA |
|  |  | 21 | 227-231 | AAAAA→AAAA |
|  |  | 24 | 386-387 | TCACCA→TCCA |
|  |  | 26 | 349 | TGGCA→TGCA |
|  |  | 27 | 363 | CGGTA→CGTA |
|  |  | 28 | 178 | ACCAG→ACAG |
| HRN *gpt*-AAI | Kidney | 29 | 289 | GGGCC→GGCC |
|  |  | 31 | 190 | CCCGT→CCGT |
|  |  | 32 | 214 | TGGCA→TGCA |
|  |  | 34 | 289 | GGGCC→GGCC |
|  |  | 35 | 226 | TGGAA→TGAA |
|  |  | 36 | 257 | ATCGC→ATGC |
|  |  | 38 | 361 | TCCGG→TCGG |
|  |  | 39 | 224 | CATGG→CAGG |
|  |  | 47 | 330 | GTTGA→GTGA |
|  |  | 48 | 347 | CATGG→CAGG |
|  |  | 50 | 166 | GGGCG→GGCG |
|  |  | 51 | 226 | TGGAA→TGAA |
|  |  | 52 | 241 | CCCAG→CCAG |
|  |  | 55 | 386-387 | TCACCA→TCCA |
|  | Liver | 6-1 | 289 | GGGCC→GGCC |
|  |  | 7-1 | 295-300 | AAAAAA→AAAAA |
|  |  | 14-1 | 289 | GGGCC→GGCC |
